# CD22 is a potential target of CAR-NK cell therapy for esophageal squamous cell carcinoma

**DOI:** 10.1186/s12967-023-04409-8

**Published:** 2023-10-10

**Authors:** Tingdang Liu, Ximing Dai, Yien Xu, Tian Guan, Liangli Hong, Tahir Zaib, Qi Zhou, Ke Cheng, Xiaoling Zhou, Changchun Ma, Pingnan Sun

**Affiliations:** 1 Stem Cell Research Center, Shantou University Medical College, Shantou, 515041 Guangdong Province China; 2 Cancer Hospital, Shantou University Medical College, Shantou, 515041 Guangdong Province China; 3 Department of Pathology, The First Affiliated Hospital of Shantou University Medical College, Shantou, 515041 Guangdong Province China; 4 Guangdong Procapzoom Biosciences, Inc., Shantou, 515041 Guangdong Province China

**Keywords:** Esophageal squamous cell carcinomas, CD22, CAR-NK cell therapy, Immunotherapy, Solid tumor

## Abstract

**Background:**

Chimeric antigen receptor NK (CAR-NK) cell therapy is one of the most promising immunotherapies. Although it has shown a significant therapeutic effect in hematologic malignancies, few successes have been obtained in solid tumors including esophageal squamous cell carcinoma (ESCC). The major reasons are lack of specific cell surface antigens and complex tumor microenvironment. Here we identify CD22, a well-known tumor surface marker in hematologic malignancies, is expressed in ESCC, possibly serving as a potential target of CAR-NK cell therapy.

**Methods:**

The expression of 13 tumor cell surface antigens used clinically was analyzed in patients from The Cancer Genome Atlas (TCGA) database. Also, mRNA expression were detected in 2 ESCC cell lines and 2 patients samples by qCPR. Then according to Venn diagram, CD22 was selected for further investigation. Following this, the expression of CD22 by immunofluorescence (IF) in ESCC cell lines and by immunohistochemistry (IHC) in 87 cases of human ESCC samples was detected respectively. On the basis of H-score results, the correlation between CD22 expression and clinical parameters was analyzed. As a proof, the efficacy of CD22-targeted CAR-NK cells against ESCC cell lines was performed by a real-time cell analyzer (RTCA) platform.

**Results:**

KYSE-140 and KYSE-150 cell lines displayed surface expression of CD22. IHC showed an 80.46% (70/87) positive rate in ESCC patient samples. Among these, cell membranous expression of CD22 was observed in 27.59% (24/87) patient samples. Through chi-square test, expression of CD22 in ESCC was associated with lymph node metastasis while it was no related to the depth of tumor invasion and clinical stage. Engineered CD22-targeted CAR-NK cells exhibited inhibitory growth capability against ESCC cell lines (*p* < 0.0001).

**Conclusions:**

CD22 is a potential tumor surface antigen capable of being targeted by CAR-NK cells in ESCC. And potential therapeutics for ESCC may be developed based on immune cells expressing anti-CD22 CAR. The study also indicates that CD22 CAR-NK cells could be used in other cancers and more in vivo experiments are needed.

**Supplementary Information:**

The online version contains supplementary material available at 10.1186/s12967-023-04409-8.

## Introduction

Esophageal cancer is one of the most common and deadly cancers. The IARC (International Agency for Research on Cancer) announced that the incidence of esophageal cancer ranks 7th and the mortality ranks 6th in the world [1]. Most esophageal cancer can be categorized into esophageal squamous cell carcinoma (ESCC) and esophageal adenocarcinoma (EAC). In Asia, ESCC typically accounts for more than 90% of esophageal cancer. In 2016, the number of newly diagnosed Chinese patients with ESCC was 252,500 and about 193,900 patients died, making ESCC the 6th most common cancer and the 5th most lethal cancer in China [2]. Clinically, most patients with ESCC are diagnosed at an advanced stage, who has poor survival. The therapeutic efficacy and prognosis of traditional treatment, especially in combination with surgery, radiotherapy and chemotherapy, remain insufficient because overall survival (OS) reaches a plateau [3]. Thus, exploring alternative treatments, such as chimeric antigen receptor (CAR)-based therapy, for ESCC is essential and of great importance.

Among CAR-based therapies, chimeric antigen receptor T (CAR-T) cell therapy has revolutionized the field of cancer treatment. For example, YESCARTA was officially approved by the U.S. Food and Drug Administration (FDA), and has been shown to be effective against hematological malignancies clinically [4, 5]. As the next generation of CAR-based therapies, chimeric antigen receptor NK (CAR-NK) cell therapy has also shown great potential [6]. It can help improve patients’ quality of life due to the remission of cancer [7].The basic framework of CAR engineered cells includes extracellular recognition domains, transmembrane domains and intracellular signal transduction domains. As the first and most important step, the extracellular recognition domains of engineered cells will bind to the tumor surface antigens. However, currently the number of tumor surface antigens suitable for CAR-based therapy is limited, especially in ESCC, which severely hinders the future application of this technology. Here, we screened for potential tumor antigens from The Cancer Genome Atlas (TCGA) database. By using this strategy, we discovered CD22 may be a potential target for ESCC.

CD22 is a transmembrane glycoprotein with a relative molecular weight of 140 kDa, and belongs to the sialic acid-dependent adhesion molecules of the immunoglobulin family [6, 8]. In B cells, CD22 is initially expressed in the cytoplasm of B cell precursors, then migrates to the cell surface, but is lost during B-cell differentiation into plasma cells. Currently, the most common target antigen for CAR based therapy in hematologic tumors is CD19. Similar to CD19, CD22 is expressed on B-cell acute lymphoid leukemia (B-ALL) cells. A phase I trial has shown the safety and efficacy of anti-CD22 CAR T cells [9]. It was widely used clinically due to its limited side effects in patients. As an attractive target in hematological malignancies, we report here that CD22 is an ESCC tumor antigen. Although induced pluripotent stem (iPS) cell-derived CAR-NK cells have made great progress in antitumor ability in some kinds of cancer [10], there are few clinical trials involving CAR-NK cell therapies in solid tumors, including ESCC. We show iPS cell-derived CD22 CAR-NK cells efficiently kill ESCC cells in vitro, which provides proof of principle that CD22 is a potential target for ESCC CAR-NK cell treatment. It is possibly a novel approach to improving the therapeutic options and prognosis for patients with ESCC on the basis of this safe target and expanding the clinical use of CD22. Besides this, it needs more in-depth in vivo experiments, such as animal experiments.

## Materials and methods

### Patients and tumor samples

A total of 87 ESCC tissue samples and para-cancerous tissues were collected from patients. All patients underwent radical resection of esophageal cancer from 2015 to 2017 in the Cancer Hospital of Shantou University Medical College after being endoscopically diagnosed pathologically. The diagnose criteria was according to the 8th edition of the American Joint Committee on Cancer (AJCC) staging of epithelial cancers of the esophagus. 63 ESCC patients were at stage III–IV due to deeper of tumor invasion and more lymph node metastasis while 24 at stage I–II. We included these patients who had not received chemotherapy or radiotherapy before surgery due to possible expression changes of targets. There are 20 females and 67 males involved in our study and all patients are aged from 44 to 76 years old. This study was approved by the Ethics Committee of Shantou University Medical College (No. 201735), and informed consent was obtained from each patient.

### Bioinformatics analysis

We acquired RNA-sequencing data of ESCC from the TCGA database, including gene expression in ESCC and corresponding clinical data. By using data from Ensemble (https://uswest.ensembl.org/index.html), we re-annotated the genetic names of the original data and obtained the gene counts for 1 normal esophagus and 81 cases of ESCC. The gene counts were calibrated and standardized using the edgeR package (http://www.bioconductor.org/) version 3.34.0 in R language version 4.0.2 (https://cran.r-project.org/). Then heat maps of gene expression were constructed.

### Cell culture

KYSE-140 and KYSE-150 esophageal cancer cell lines were cultured in RPMI medium (SH30809.01, HyClone) containing 10% newborn bovine serum (Gibco) and antibiotics (Pen/Strep, 15,140–122, Gibco), whereas the 293T and K562 cell lines were cultured in high glucose dulbecco’s modified eagle medium (DMEM) containing 10% newborn bovine serum and antibiotics. All the cells were cultivated in a incubator at 37 ℃ and 5% CO_2_. When digesting cells, 0.05% trypsin–EDTA (25,200,072, eBioscience) was used.

### RNA extraction and quantitative polymerase chain reaction

Total RNA was extracted from cell lines (KYSE140, KYSE150) and two ESCC patients’ tissues using RNAiso (RNAiso Plus, 9109, Takara), and cDNA was synthesized using a ReverTra Ace qPCR RT Kit (FSQ-101, TOYOBO). Quantitative polymerase chain reaction (qPCR) was performed with qPCR SYBR Green Master Mix (A25742, Thermo Fisher Scientific). The expression level of target genes was normalized to GAPDH. RT-qPCR data were analyzed by the 2^-ΔΔCT method. The primer sequences used are listed in Additional file 1. The information of two patients involved in the qPCR analysis is as follows: Patients-1 and patient-2 were both male and at stage III. Patient 1 was 58 years old and patient 2 was 59 years old.

### Immunofluorescence

KYSE140 and KYSE150 cell lines were fixed in cold methanol for 30 min, washed with PBST (PBS, ZLI-9063, ZSGB-BIO; Tween, V900548, VETEC), and blocked with 2% BSA for 60 min at room temperature, then incubated with primary CD22 antibody (1:200; ab207727, Abcam) at 4 ℃ overnight, followed by washing and incubation with 1:1000 Alexa Fluor 488-conjugated fluorescent secondary antibodies (ZF-0511, ZSGB-BIO) for 2 h at room temperature in a dark environment. Cells were counterstained with DAPI (diluted with water at a ratio of 3:7, C1006, Beyotime) and analyzed by fluorescence microscopy (Axio Observer A1-Zeiss).

### Immunohistochemistry

Expression of CD22 in 87 ESCC samples (formalin-fixed and paraffin-embedded) was detected using immunohistochemistry (IHC). ESCC and tonsil sections (positive control) were subjected to citrate antigen retrieval solution. Added the citrate antigen retrieval solution to the pressure cooker and boiled the slices for 10 min. Normal equine serum (1:10, ZL-9024, ZSGB-BIO) was used to prevent non-specific staining for 1 h at 37 ℃. Then, sections were incubated with primary anti-CD22 antibody at a 1:100 dilution in PBS overnight for 14 h at 4 ℃, followed by a two-step detection kit including secondary antibody (PV-9000, ZSGB-BIO). DAB was for color development (ZLI-9018, ZSGB-BIO), followed by counterstaining with hematoxylin (3,201,111, Wexis). According to the location of the DAB signal, we can judge where it expresses.

### Concentration dependent lysis efficiency of NK cells measuring

K562 cells were seeded in the 96-well plate for 1 × 10^4^ cells in each well with K562 cell culturing medium. For the lysis efficiency of NK cells measurement, NK cells were added in the wells in different ratios of NK cells: K562 cells from 8: 1 to 1: 2 and co-culturing for 12 h. K562 cells were cultured with NK culturing medium treating as the basement. Then the tumor cells viabilities were analyzed with the CCK-8 assay kit (C0039, Beyotime Biotech, China) after washing out NK cells. The killing rate was calculated as the formula: 100% − (the viability of each group/the viability of the basement) × 100%.

### Derivation of natural killer cells

The human UKKi011-A iPS cell line (66540010, Sigma) was maintained according to the instructions. To produce mature and functional natural killer (NK) cells from iPSC lines, we used a previously published protocol with minor changes [11, 12]. In brief, 6000 TrypLE-adapted iPSCs were seeded in 96-well low attachment plates in BPEL (bovine serum albumin, polyvinyl alcohol, essential lipids) containing 40 ng/mL stem cell factor (SCF) (R&D Systems), 20 ng/mL vascular endothelial growth factor, and 20 ng/mL bone morphogenic protein 4 (R&D Systems). On Day 11 of hematopoietic differentiation, embryoid bodies (EBs) were directly transferred into each well of uncoated 24-well plates. Cells were then further differentiated into NK cells using 5 ng/mL IL-3, 10 ng/mL IL-15, 20 ng/mL IL-7, 20 ng/mL SCF), and 10 ng/mL Flt3 ligand (R&D Systems) for 30 days. As described above, our major improvement was transducing the CD22-CAR into the iPS cells first, and then inducing the CD22-CAR-iPS cells to differentiate into CD22-CAR-NK cells. This strategy efficiently raised the living ratio of the CAR-NK cells and reduced the suffering damages or toxicities from transduction regents for CAR-NK cells.

### iPS cell-derived CD22 CAR-NK cells development

We constructed CD22-CAR with a sequence including a CD22-targeting scFv sequence, a 4-1BB sequence and a CD3ζ signal peptide. The sequences were connected with a transmembrane domain and were transfected in HEK293T cells utilizing the pWXLD lentiviral vector embedded in lentivirus. Then the CD22-CAR sequence was extracted from the HEK293T cells with a kit (AG51001, Accurate Biology Inc., China) and used for iPS cells transduction. The iPS cells were transfected with viral particles containing the CD22-CAR sequence and cultured in mTeSR Plus medium (#100-0276 C, STEMCELL, Canada). Subsequently, the CD22-CAR-NK cell derivation steps were followed according to the procedures in "Derivation of natural killer cells".

### Real-time cell analyzer (RTCA) platform analysis

The cell index of KYSE140 and KYSE150 was monitored by the xCELLigence RTCA instrument (RTCA, S16, Agilent, USA) for measuring the lysis efficiency of CD22-targeted CAR-NK cells. The KYSE140 or KYSE150 cells were suspended at the concentration of 1 × 10^5^ cells/mL in the culturing medium and seeded into E-Plate 16 for 100 µL in each well. Then, the CD22-targeted CAR-NK or non-relevant NK cells were added into E-Plate 16 at the effect cells: target cells (E: T) ratio of 1: 1. The NK culturing medium was added to the well as the control group. Impedance was recorded every 15 or 20 min until the test finished. The final results were plotted with GraphPad Prism (version 8, GraphPad Software, USA) and analyzed with the Two-way-ANOVA method in group analysis by GraphPad Prism.

### Statistical analysis

Clinical data were prospectively collected according to standardized protocols. To conduct statistical analyses, SPSS (Version 20, IBM) software was used. The relation between clinical data and IHC staining was analyzed through chi-square and Fisher tests, and shown in a cross-tabulation table. Survival curves were plotted with the Kaplan–Meier method and then analyzed by log-rank testing.

## Results

### Identification of CD22 as a potential target by a joint screening strategy

**Fig. 1:**
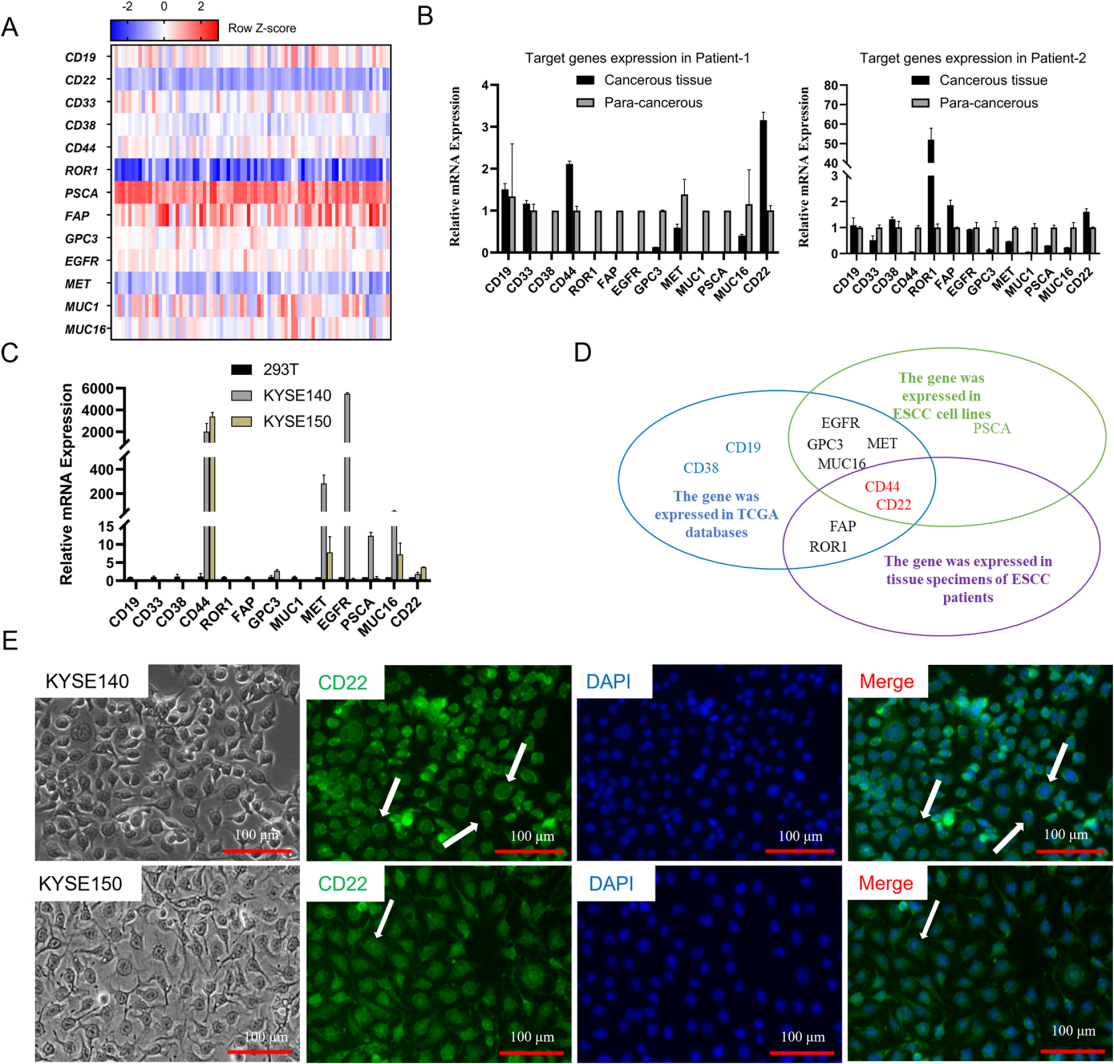
Screening and validation of CD22 expression.** A** Heat maps for the expression of 13 target genes from RNA-seq data in 1 normal esophagus and 81 cases of ESCC in TCGA. **B** Expression of the 13 target genes in 2 ESCC patients. **C** Expression of the 13 target genes in two ESCC lines (KYSE140 and KYSE150). **D** Venn diagram of the bioinformatics analysis and qPCR results. **E** CD22 expression in KYSE140 and KYSE150 cells was examined by immunofluorescence. White arrows show CD22 expressed on the cell membrane. Scale bar: 100 μm

Thirteen targets, i.e. CD19, CD20, CD22, CD33, CD38, CD44, ROR1, PSCA, FAP, GPC3, EGFR, MET, MUC1, and MUC16, were selected based on current use in clinical trials (https://clinicaltrials.gov/) in combination with the reviews of Jackson [13] and S Depil [14]. Gene expression maps were made based on TCGA data, and the expression values of the 13 target genes were extracted. The gene counts were calibrated and standardized using the edgeR package (http://www.bioconductor.org/) version 3.34.0. To facilitate the comparison of expression values, they were normalized by converting the values to log10, then drawing a heatmap based on the values obtained through the calculations (Fig. 1A). Compared with normal esophageal tissue, CD33, PSCA and MUC1 expression levels were lower. Secondly, we obtained two ESCC samples from the Cancer Hospital of Shantou University and determined whether the mRNA expression levels were similar to those in TCGA. Among CD44, ROR1, FAP and CD22, compared to adjacent tissue, at least one of the genes was highly expressed in ESCC tissue (Fig. 1B). Thirdly, characterization of the mRNA expression levels of 13 target genes in 2 ESCC cell lines (KYSE140, KYSE150) showed that among CD44, GPC3, MET, EGFR, PSCA, MUC16 and CD22, at least one of the genes was relatively highly expressed in each ESCC cell line (Fig. 1C). The Venn diagram summarizes the above data and shows that CD44 and CD22 were the most commonly shared targets (Fig. 1D). Researchers have already reported that CD44 is highly expressed in tumor initiating cells of ESCC [15]. Furthermore, CD22 is relatively highly expressed in ESCC cell lines and ESCC tissues from both patients. So, we focused on CD22 in this study, a potential new antigen target for ESCC. We further examined the expression of CD22 through immunofluorescence (Fig. 1E). Results showed that CD22 was expressed in both KYSE140 and KYSE150 cell lines, which is consistent with the qPCR results.

### CD22 is expressed in ESCC cell lines and tissue specimens of ESCC patients

KYSE140 and KYSE150 cells expressed CD22 mRNA, and in ESCC patients, mRNA expression of CD22 in esophageal squamous cell carcinoma was higher than in para-cancerous tissue (Fig. 1B, C). Ten ESCC samples and their corresponding para-cancerous tissues were detected by qPCR to verify our conclusion. The results showed that among 10 ESCC patients, mRNA expression of CD22 in esophageal squamous cell carcinoma was higher than in para-cancerous tissue in 5 patients (Additional file 3). IHC staining also indicated that normal epithelial cells in para-cancerous tissues did not express CD22 (Additional file 2). In immunofluorescence staining of ESCC cell lines, CD22 could be found in the cell membrane and cytoplasm (Fig. 1E). The membrane location of CD22 is indicated by the white arrows. Expression of CD22 in ESCC patient samples needs further exploration, since gene expression in cell lines is sometimes not consistent with tissue samples.

**Fig. 2:**
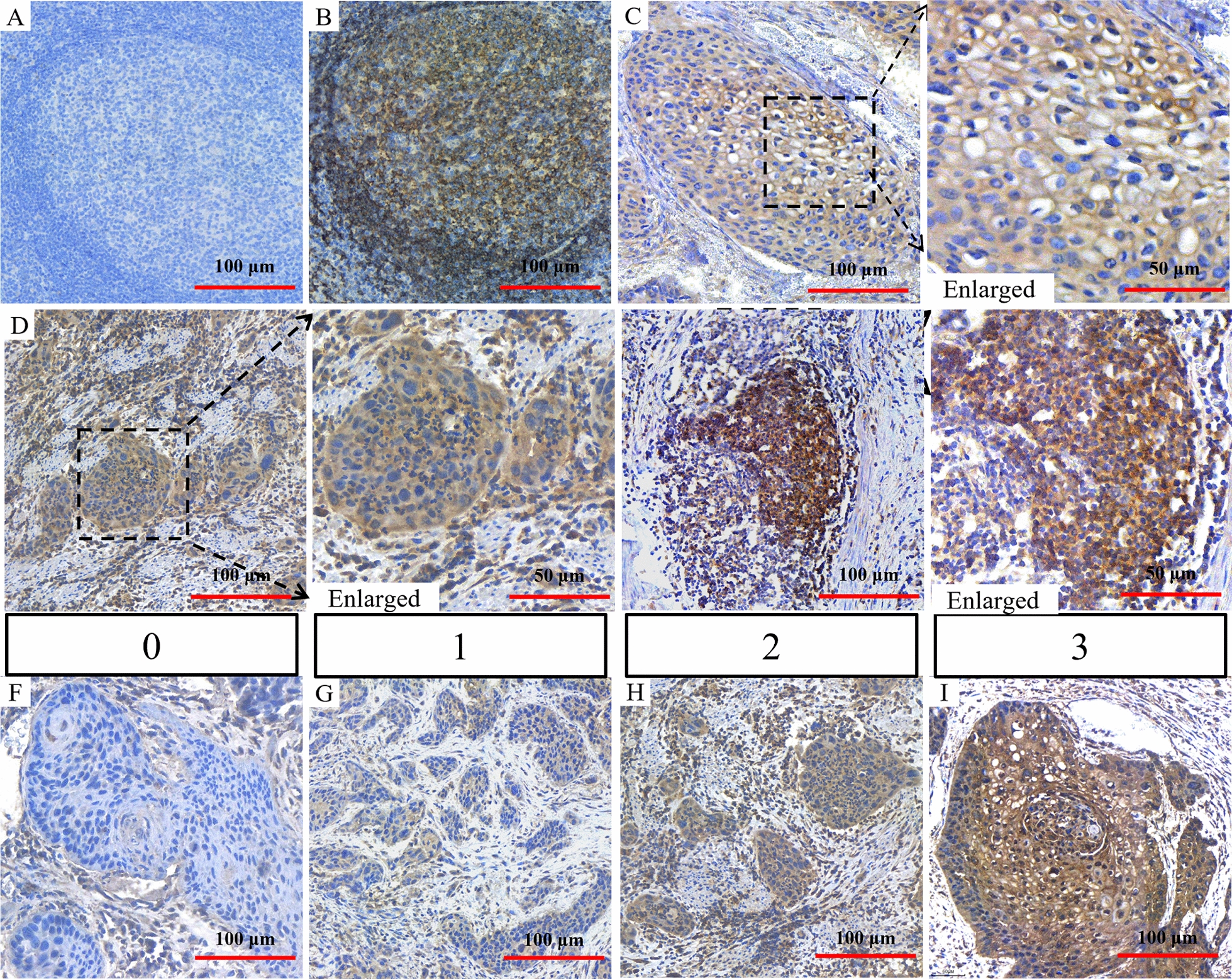
Expression of CD22 in tissue samples from ESCC patients. **A** Tonsil cancer specimens incubated with PBS without primary antibody (negative control). **B** CD22 expression in tonsil carcinoma incubated with primary antibody (positive control). **C**–**E** Expression of CD22 in ESCC patients. **C** Membranous CD22 expression. **D** Cytoplasmic CD22 expression. **E** CD22 expression in lymphocytes within the tumor. **F**–**I** Different levels of CD22 expression as related to H-score. **F** Negative CD22 expression, score 0, the tumor cell proportion score is 0%. **G** Weak-positive CD22 expression, the tumor cell proportion score is 0%, score 1. **H** Moderate-positive CD22 expression, the tumor cell proportion score is 90%, score 2. **I** Strong-positive CD22 expression, the tumor cell proportion score is 90%, score 3. Scale bar, 100 μm

We analyzed the expression of CD22 in 87 ESCC patients by IHC. Tonsil carcinoma sections incubated with PBS served as negative controls (Fig. 2A). CD22 is broadly known as a marker of B cells, so we used tonsil carcinoma which is rich of B cells as positive controls (Fig. 2B). Staining showed CD22 was strongly expressed in lymphnodules of tonsil carcinoma. In the enlarged picture, expression in the tumor cell membrane was obvious (Fig. 2C). In a second patient, CD22 was expressed mostly in the cytoplasm of tumor cells (Fig. 2D), while in some cases, CD22-positive lymphocytes were found in the stroma (Fig. 2E). As CD22 is always expressed in B cells, it should be noted that the expression of CD22 in lymphocytes within the tumor would be a good internal control for IHC staining quality.

Furthermore, subcellular localization was analyzed. In our patient samples, IHC staining showed that CD22 was expressed in 80.46% (70/87) of patients, whereas 19.54% (17/87) samples had little or no expression of CD22 in the tumor cells. In positive patients, 46 cases (52.87%) were both membrane- and cytoplasm-positive, and 24 cases (27.59%) were membrane-positive (Table 1). And we found that the tumor cell proportion rate was up to more than 60% in the majority of CD22-positive ESCC samples which indicated that CD22 was widely expressed across tumor cells. Besides this, the normal epithelium will not express CD22 (Additional file 4). This suggests CAR-NK cell therapy targeting CD22 may benefit more than half of ESCC patients.

**Table 1:** CD22 immunohistochemical staining

Location of CD22	Number	Percent
Cell membrane and cytoplasmic positive	46	52.87
Cell membrane positive	24	27.59
Cell membrane negative	17	19.54

With the help of pathologists, the H-score was assessed in tissue slices of 87 ESCC patients. Here we list the representative IHC results for different H-scores.

Staining intensity as follows: A score of 0 indicated negative or little expression in tumor cells (Fig. 2F). A score of 1 represented weak positive expression (Fig. 2G), 2 represented moderate positivity (Fig. 2H), and 3 represented highly positive (Fig. 2I) expression on slices. The percentage of positive cells as follows: 0 indicated the positive tumor cell proportion ranged from 0 to 10%; 1 indicated the positive tumor cell proportion ranged from 10 to 25%; 2 indicated the positive tumor cell proportion ranged from 26 to 50%; 3 indicated the positive tumor cell proportion ranged from 51 to 75%; 4 indicated the positive tumor cell proportion ranged from 75 to 100%. The H-score was the sum of staining intensity and the percentage of positive cells.

### Relationship between CD22 expression and clinical parameters

We further analyzed the expression of CD22 and its correlation with clinical parameters (Table 2). Interestingly, ESCC patients with different CD22 expression levels had statistically significant differences in lymph node metastasis (*p* < 0.05, *p* = 0.008), suggesting that CD22 may promote cancer cell lymph node infiltration. No correlations were found between CD22 expression and gender (*p* > 0.05, *p* = 1.000), age (*p* > 0.05, *p* = 0.592), depth of tumor invasion (*p* > 0.05, *p* = 0.137) and clinical stage (*p* > 0.05, *p* = 0.546).

**Table 2:** CD22 expression levels and clinical parameters in ESCC tissues (n = 87)

Clinical parameters	CD22						
	Total		Expression				*P-*value
	n	%	Negative		Positive		
			n	%	n	%	
Total number	87	100.00	17	19.54	70	80.46	
Sex							1.000
Female	20	22.99	4	20.00	16	80.00	
Male	67	77.01	13	19.40	54	80.60	
Age							0.592
≤ 59	41	47.13	9	21.95	32	78.05	
≥ 60	46	52.87	8	17.39	38	82.61	
Depth of tumor invasion							0.137
pT1–pT2	14	16.09	5	35.71	9	64.29	
pT3–pT4	73	83.91	12	16.44	61	83.56	
Lymph node metastasis							0.008
Negative	32	36.78	11	34.38	21	65.63	
Positive	55	63.22	6	10.91	49	89.09	
Clinical stage							0.546
I–II	24	27.59	6	25.00	18	75.00	
III–IV	63	72.41	11	17.46	52	82.54	

### CD22 expression and correlation with overall survival in ESCC

**Fig. 3:**
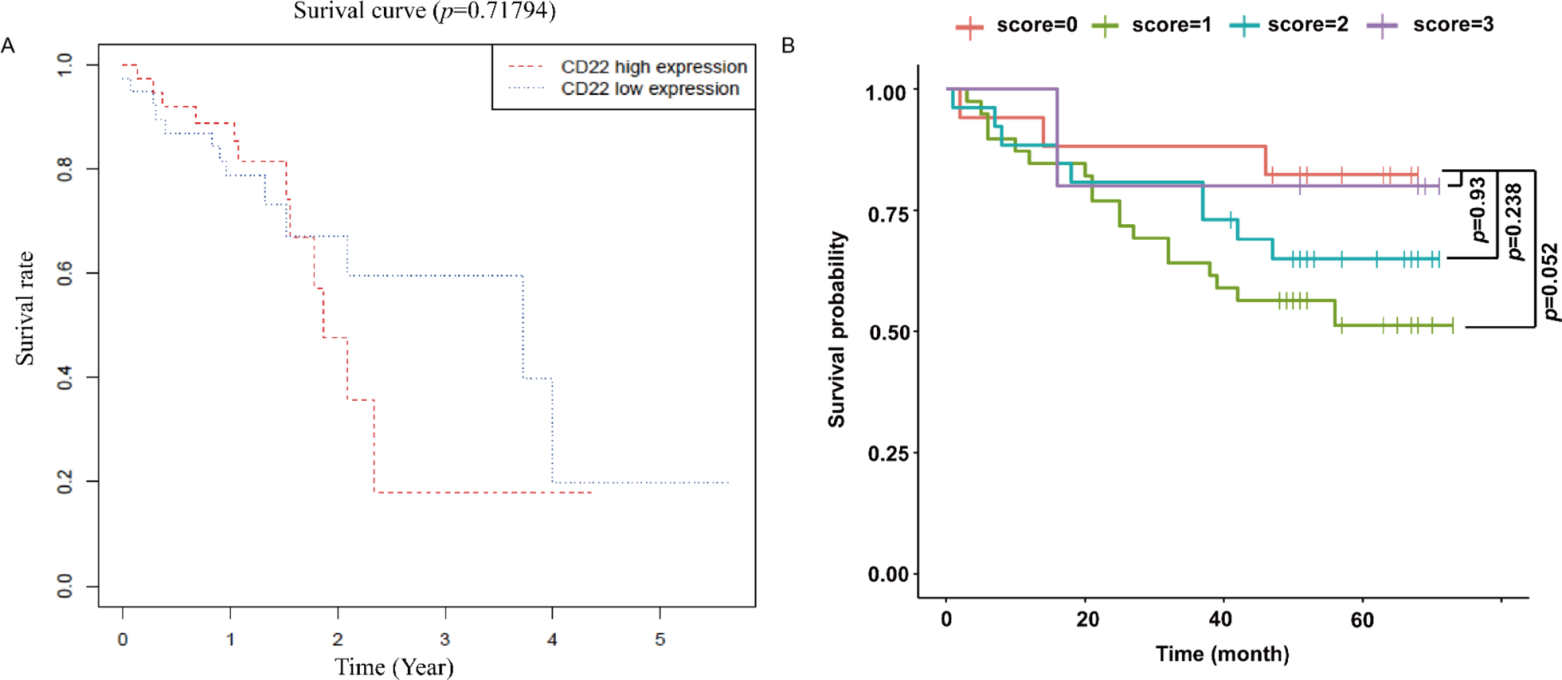
Kaplan–Meier survival analysis. **A** Overall survival analysis based on ESCC clinical data from TCGA. **B** CD22 expression and overall survival analysis based on 87 cases of esophageal squamous cell carcinoma. No correlation between OS and CD22 expression was observed

Kaplan–Meier survival curves based on ESCC patient CD22 expression in TCGA were drawn (Fig. 3A). Additionally, Kaplan–Meier survival curves were made on the basis of 87 patients with ESCC from the Cancer Hospital of Shantou University Medical College (Fig. 3B). As shown in Fig. 3A, CD22 expression was not related to the overall survival (OS) of patients from TCGA. Figure 3B shows that the OS of our patient cohort from the Cancer Hospital of Shantou University Medical College, regardless of CD22 positivity, was the same as those who were CD22-negative.

### CD22 iPS-CAR NK cells show strong anti-tumor activity against ESCC cell in vitro

**Fig. 4:**
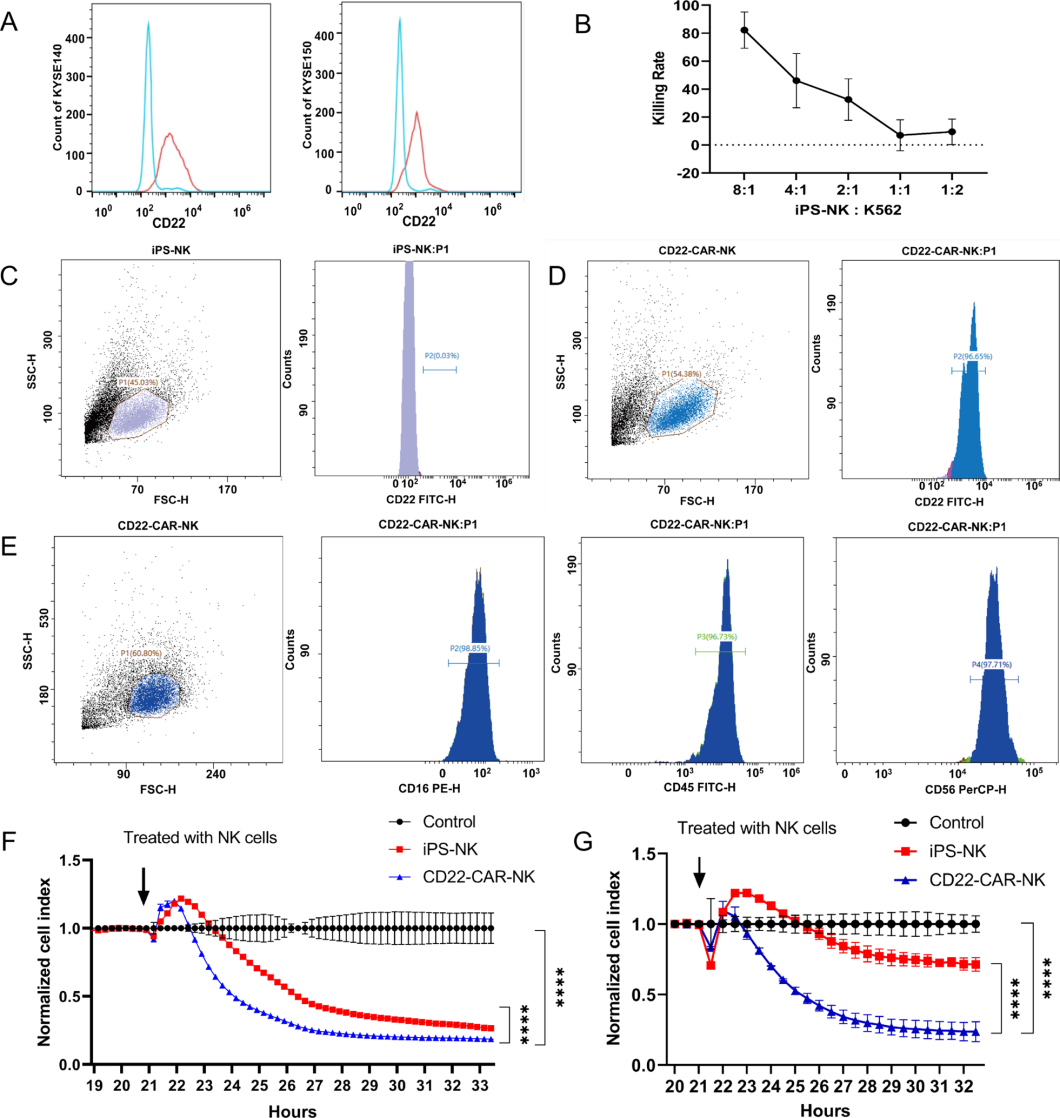
CD22 iPS-CAR NK cells showed anti-tumor activity against CD22-positive ESCC cell lines in vitro. **A** CD22 expression in KYSE140 and KYSE150 was examined by flow cytometry. The majority of KYSE-140 and KYSE-150 cells had CD22 expression (red outline) compared with a control group (without primary antibody, blue outline). **B** Concentration-efficacy curve of iPS-NK-mediated killing of K562 cells. **C** CD22-CAR was not expressed in NK cells derived from iPS (iPS-NK) but expressed obviously in CD22-CAR-NK cells. **D** High CD22 expression in CD22-CAR-NK cells. **E** CD22-CAR-NK cells express specific NK cell markers, including CD16, CD45 and CD56. **F** Time-cell index curve of CD22-CAR-NK-mediated killing of KYSE140 cells. Data represent the mean ± SEM of ten independent experiments. ****p < 0.0001. **G** Time-cell index curve of CD22-CAR-NK cells killing KYSE150 cells. Data represent the mean ± SEM of ten independent experiments. ****p < 0.0001

The membranous expression of CD22 in KYSE140 and KYSE150 cells was further analyzed by flow cytometry, which confirmed membranous expression of CD22 (Fig. 4A). The gating strategy is listed in Additional file 5. Next, a previously published protocol was used to obtain NK cells and verified their killing ability against K562 cell line [11] (Fig. 4B). The results showed that the killing rate of iPS-NK cells increased along with increasing numbers of iPS-NK cells. Flow cytometry showed a single-chain variable fragment of CD22 is highly expressed in CD22-CAR-NK cells but not in iPS-NK cells (Fig. 4C, D). At the same time, CD22-CAR-NK cells also expressed NK cell markers (Fig. 4E). Finally, the engineered CD22-targeted CAR-NK cells were used to co-culture with two ESCC cell lines respectively and observed the following changes by xCELLigence system. Clearly, CD22-CAR-NK cells displayed higher cytotoxicity compared with iPS-NK cells, as shown by the cell index of KYSE140 and KYSE150 cells decreasing more quickly (Fig. 4F, G). These results indicate that CD22 iPS-CAR NK cells possessed the potential function to kill most esophageal squamous cells.

## Discussion

Nowadays, CAR therapy has shown success in hematological malignancies, but has met with major obstacles in the treatment of solid tumors due to lack of specific antigens [16]. In ESCC treatment, tumor-associated targets applied clinically are rare but have included EGFR, VEGF, HER-2 and PD-L1 [17, 18]. As for CAR therapy in ESCC, few targets have been found in clinical trials (ClinicalTrials.gov). In order to find a new target for CAR-treatment of ESCC, we hypothesized that CAR-associated targets now used in clinical trials, even clinically, are relatively safe, and we determined whether any of them could be used for treatment of ESCC. We found that CD22 is highly expressed in ESCC, suggesting potential use for current CD22 CAR therapies in the treatment of ESCC.

In this study, we selected 13 targets from CAR-related clinical trials for further analysis. From the Venn diagram, based on data in TCGA as well as cell lines and ESCC specimens, we found that CD22 and CD44 are possibly expressed in ESCC. CD44 is well-known to be expressed in ESCC, thus CD22 provides a second option and has never been found in solid tumors, particularly ESCC. We further performed IHC to determine the expression of CD22 in 87 ESCC patient samples. Results showed that CD22 is widely over-expressed in ESCC (80.46%), with membranous expression clearly observed in 24 (27.59%) of 87 ESCC tissues. And the tumor cell proportion rate is up to more than 60% in the majority of CD22-positive ESCC samples which indicates that CD22 is widely expressed across tumor cells. The relationship between expression and clinical parameters reveals that CD22 may contribute to lymph node metastasis, but is not related to depth of tumor invasion or clinical stage (*p* > 0.05, respectively). Despite that, CD22 expression does not influence ESCC patients’ overall survival. We conclude that one reason may be insufficient IHC samples. Another reason is that lymph node matastasis do not always correlate with survival rate as reported [19]. We will analyze more samples in the future and maybe it can reveal confounding factors. The study suggests that CD22 is expressed in ESCC patients. For the location of CD22, flow cytometry and IF also reveal that CD22 is expressed on the cell surface, thus enabling it to serve as a potential target in CAR-NK cell therapy. We show CD22-target CAR-NK cells inhibit the growth of ESCC cell lines, suggesting CD22 CAR-NK cell therapy is a potential method for ESCC.

Expression of CD22 in solid tumors included lung cancer. Tuscano et al. found high expression of CD22 in lung cancer cells, but was not reproducible by Pop LM [20, 21]. What’s more, our group has published another paper to indicate CD22 expression in breast cancer [22]. Our study found that CD22 was widely expressed in the ESCC cell membrane. Based on the relationship between CD22 expression and clinical parameters, CD22 in esophageal squamous cells may contribute to lymph node metastasis.

It is broadly known that CAR-NK cell therapy has more advantages than CAR-T cell therapy. In July 2017, Novartis’ CAR-T cells were officially approved by the FDA. Besides this, CD22 CAR-T cell therapy has been shown to be effective against hematological malignancy in previous clinical studies [23]. However, the risk of cytokine release syndrome, graft-versus-host disease (GVHD), neurotoxicity, on-target off-tumor effects and other adverse reactions restrict the clinical applications of CAR-T cell therapy. Compared to CAR-T cell therapy, the risk of on-target off-tumor effect is lower because of limited lifetime of CAR-NK cells in circulation [24]. GVHD can be reduced by allogeneic CAR-NK cell infusion [25]. NK cells do not secrete inflammatory factors that cause cytokine release syndrome, such as IL-1 and IL-6, whereas T cells do [26, 27]. In addition, NK cells can be obtained from a wide range of sources, including peripheral blood, umbilical cord blood, induced pluripotent stem cells, NK-92 and other cell lines [10, 28, 29]. What’s more, it still have the potential drawbacks, for instance, could include the difficulty in manufacturing CAR-NK cells, the potential for off-target effects, or the immunogenicity of CAR-NK cells. In general, engineered CAR-NK cells show great potential.

From the advantages mentioned above, CD22 CAR-NK cells might exert good tumor cytotoxicity in ESCC treatment. Based on this, we constructed CD22 CAR-NK cells, then testing their ability to kill tumor cells in vitro. CD22 iPS-CAR NK cells showed antitumor activity against CD22-positive ESCC cell lines. So we infer that clinically, CD22 CAR-NK cells may have efficacy in treating ESCC, but more in-depth exploration is required. If CD22 CAR-NK cells cannot induce remission in ESCC patients, constructing a dual-antigen CAR may result in synergistic responses in solid tumors [30, 31]. CD44 is highly expressed in ESCC [15] and in our experiments, we assumed that the construction of a dual CD44/CD22 CAR drug would have a better effect in treating ESCC.

As a target in CAR therapy, CD22 has been shown to be relatively safe for patients, but the possibility of toxicity of CD22-CAR-NK cell therapy in ESCC must be considered. Human CD22 is normally restricted to B-cells [32]. The location of CD22 is on the cell membrane which indicates that it is a good target for treating cancer by CAR-T or CAR-NK cell therapy, preventing off-target effects. Pan announced that CD22 CAR-T cell therapy was used in patients with refractory or relapsed B acute lymphoblastic leukemia, and the patients only suffered mild cytokine release syndrome and neurotoxicity related to CAR-T cell therapy [23]. In our study, IHC showed CD22-positive lymphocytes in the stroma, which indicates that these lymphocytes may also be attacked by CAR-NK cells during treatment. CD22-targeted CAR-NK cells could have off-target effects, considering CD22 is a B cell marker. However, we believe that CD22 CAR-NK cell therapy will be effective in the treatment of ESCC with limited side effects, but it need exploration.

Additionally, CD22-targeting CAR-NK cells in combination with nanomaterials can induce synergistic antitumor ability which offers a new way to enhance efficacy. NK cells’ ability can be strengthened by nanoparticles [33–35]. As a carrier, it can deliver drugs to the target area which helps prevent on-target off-tumor effect [36, 37]. Kwang-Soo Kim et al. induced higher cytotoxicity against triple-negative breast cancer by using polyethyleneimine coated-cationic iron oxide nanoparticles [38]. In brief, it can be used in the delivery of CD22 CAR-NK towards tumor cells.

## Conclusions

In summary, we report the development of a CAR-NK cell directed against CD22, that shows strong activity against ESCC, making it a potentially new therapeutic option for patients with ESCC. Due to the limitations of the methods used, such as the sensitivity, specificity, or reproducibility of IHC employed for CD22 detection, we need to combine more methods to confirm the conclusion. In the future, more pre-clinical studies are needed to confirm the efficacy and side effects of CD22 CAR-NK cells. And we must do in vivo experiments like humanized mice [39] before these CAR cells can be used in ESCC patients. What’s more, its mechanisms need exploration and longitudinal studies should be performed to understand whether CD22 expression changes over time and how this impacts treatment efficacy.

### Supplementary Information

**Additional file 1. **Primer Sequences.

**Additional file 2. **Expression of CD22 in ESCC tissues.

**Additional file 3. **Expression of CD22 in ten ESCC patients.

**Additional file 4. **CD22 expression in ESCC patients' corresponding para-cancerous tissue.

**Additional file 5. **Expression of CD22 in ESCC cell lines.

## Data Availability

Data is available from the authors upon request. Thirteen targets which are current used in clinical trails can be found at: http://clinicaltrails.gov. Gene expression maps RNA-seq data was acquired from TCGA database: https://www.genome.gov/Funded-Programs-Projects/Cancer-Genome-Atlas. Related gene names was transformed according data from Ensemble: https://uswest.ensembl.org/index.html.

## Abbreviations

CAR-NK: Chimeric antigen receptor NK
ESCC: Esophageal squamous cell carcinoma
TCGA database: The Cancer Genome Atlas database
IF: Immunofluorescence
IHC: Immunohistochemistry
IARC: International Agency for Research on Cancer
EAC: Esophageal adenocarcinoma
CAR: Chimeric antigen receptor
CAR-T: Chimeric antigen receptor T
FDA: U.S. Food and Drug Administration
B-ALL: B-cell acute lymphoid leukemia
iPS: Induced pluripotent stem
DMEM: Dulbecco’s modified eagle medium
qPCR: Quantitative polymerase chain reaction
NK: Natural killer
BPEL: Bovine serum albumin, polyvinyl alcohol, essential lipids
SCF: Stem cell factor
EBs: Embryoid bodies
OS: Overall survival
GVHD: Graft-versus-host disease
