# Supplementary material for: CD22 is a potential target of CAR-NK cell therapy for esophageal squamous cell carcinoma
Source: J Transl Med. 2023 Oct 10;21:710. doi: 10.1186/s12967-023-04409-8 (PMC10563326; doi:10.1186/s12967-023-04409-8)
Supplement: Supplementary file 1 — Additional file 1. Primer Sequences. [file 12967_2023_4409_MOESM1_ESM.docx]

**Additional Files 1**

**Additional File 1. Primer Sequences**

| Gene name | Primer(3’-5’) | Product(bp) |
| --- | --- | --- |
| *GAPDH*-F  *GAPDH*-R  *CD19*-F  *CD19*-R  *CD22*-F  *CD22*-R  *CD33*-F  *CD33*-R  *CD38*-F  *CD38*-R  *CD44*-F  *CD44*-R  *ROR1*-F  *ROR1*-R  *FAP*-F  *FAP*-R  *EGFR*-F  *EGFR*-R  *GPC3*-F  *GPC3*-R  *MET*-F  *MET*-R  *MUC1*-F  *MUC1*-R  *PSCA*-F  *PSCA*-R  *MUC16*-F  *MUC16*-R | ACAACTTTGGTATCGTGGAAGG  GCCATCACGCCACAGTTTC  GGCTATGAGGAACCTGACAGTG  TCATCCTCAGGGTTCTCGTAGC  GCACCCTGAAACCCTCTACG  ATCAAACTTCGAGGTGTTCTTGT  GGCCACTCCAAAAACCTGAC  GACAACCAGGAGAAGATCGGG  CAACTCTGTCTTGGCGTCAGT  CCCATACACTTTGGCAGTCTACA  CTGCCGCTTTGCAGGTGTA  CATTGTGGGCAAGGTGCTATT  CAGTCAGTGCTGAATTAGTGCC  TCATCGAGGGTCAGGTAAGAAT  ATGAGCTTCCTCGTCCAATTCA  AGACCACCAGAGAGCATATTTTG  AGGCACGAGTAACAAGCTCAC  ATGAGGACATAACCAGCCACC  CCTTTGAAATTGTTGTTCGCCA  CCTGGGTTCATTAGCTGGGTA  ACTGAGAGGCTCCGAGAAATG  GAACCCCGCATCTTGGCTT  CGCTGCGACACTACATCAAC  CTCTGGGCTGGATCGTTTTCC  TGCTGCTTGCCCTGTTGAT  CCTGTGAGTCATCCACGCA  CCAGTCCTACATCTTCGGTTGT  AGGGTAGTTCCTAGAGGGAGTT | 101  113  136  77  193  109  82  215  177  217  103  62  216  161 |

**Additional File 2. Expression of CD22 in ESCC tissues.**


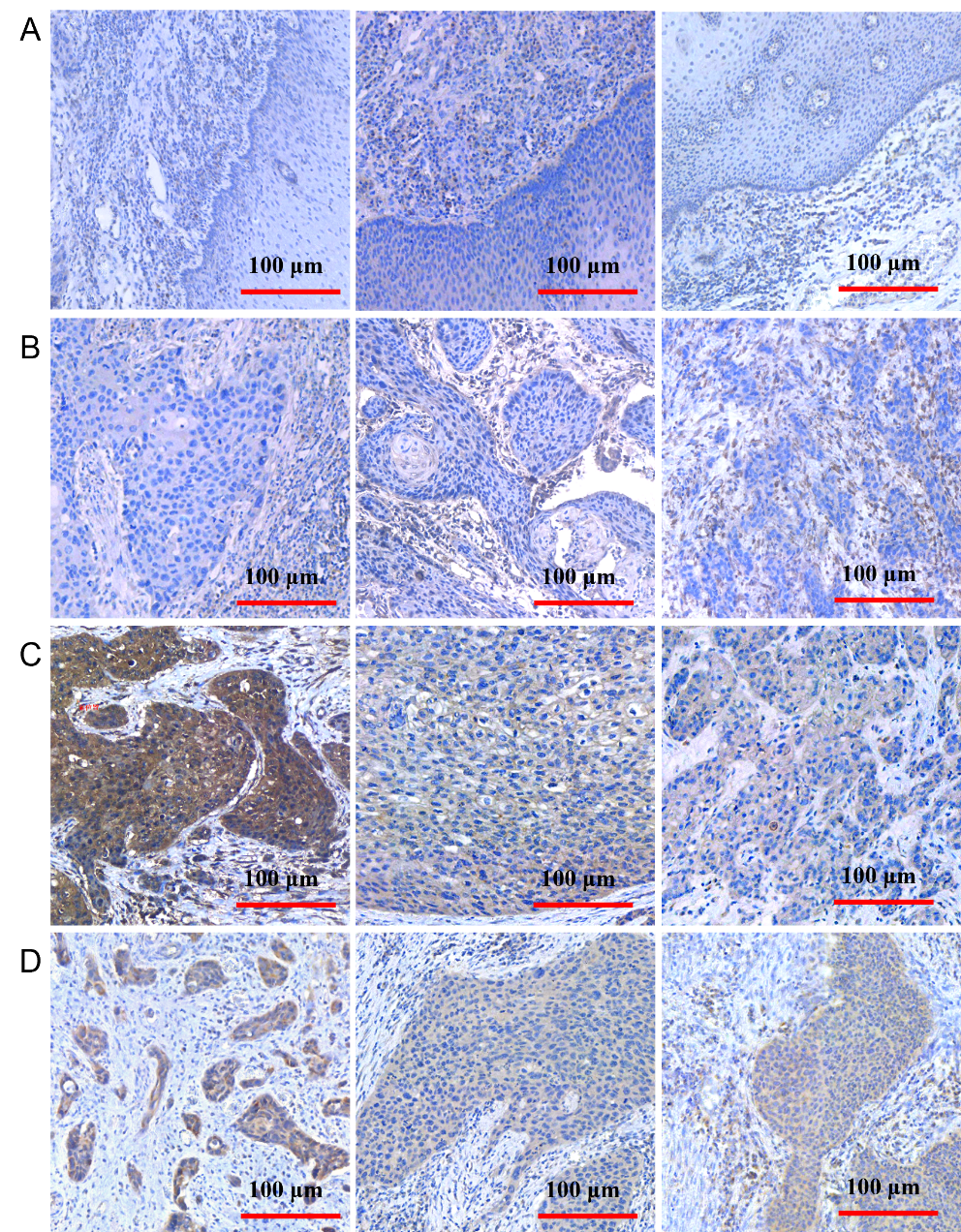


**Additional File 2. Expression of CD22 in ESCC tissues.**

A. Negative CD22 expression in para-cancerous tissue

B. Negative CD22 expression in cancerous tissues

C. Positive cell membrane CD22 expression

D. Positive CD22 expression in the cytoplasm

Scale bar, 100 μm.

**Additional File 3. Expression of CD22 in ten ESCC patients**


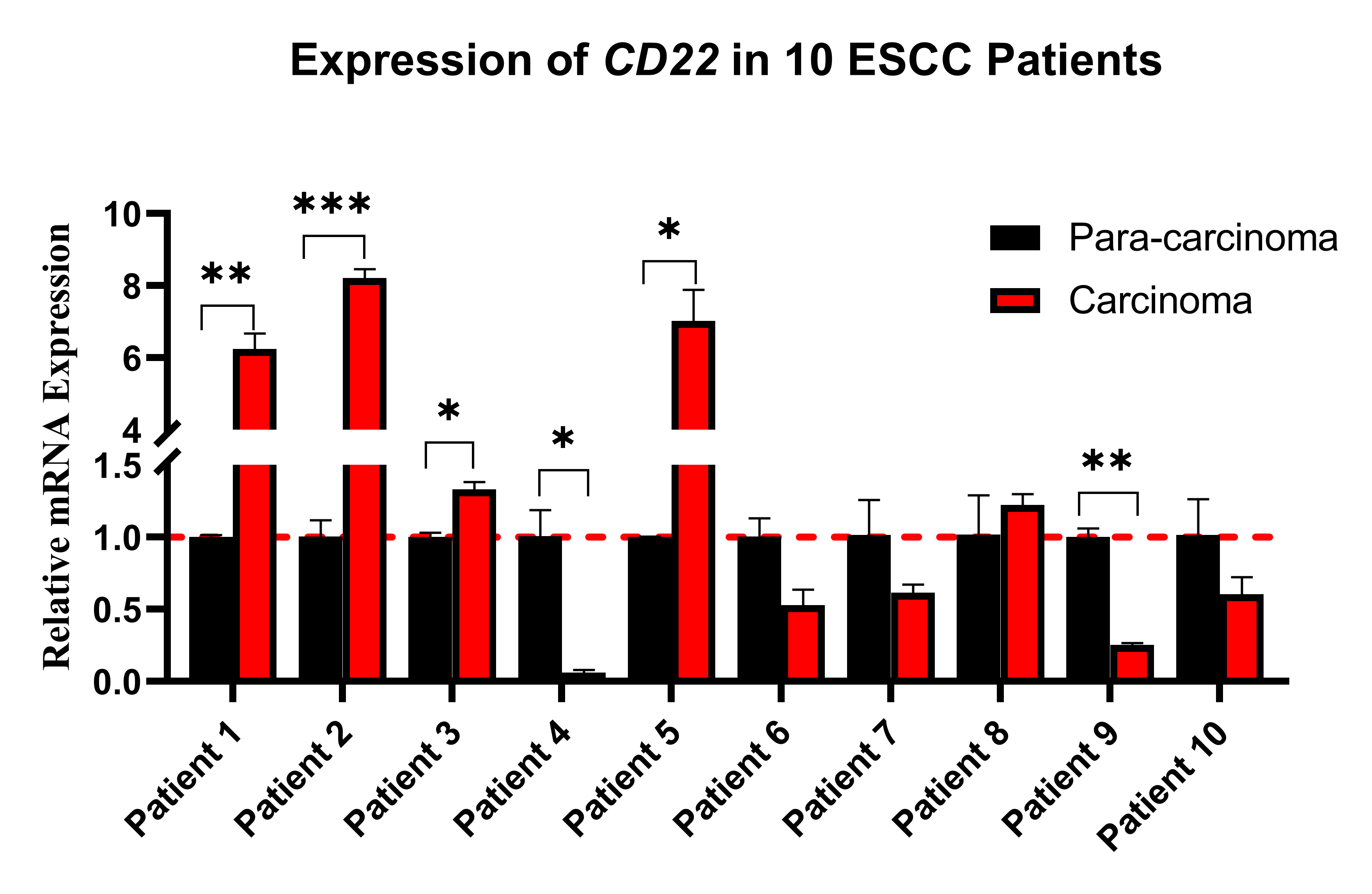


**Additional File 3. Expression of CD22 in ten ESCC patients**

Expression of CD22 in ten ESCC patients’ cancerous tissues and theirs’ corresponding para-cancerous tissues. *p < 0.05, **p < 0.01, ***p < 0.001.

**Additional File 4. CD22 expression in ESCC patients' corresponding para-cancerous tissue**


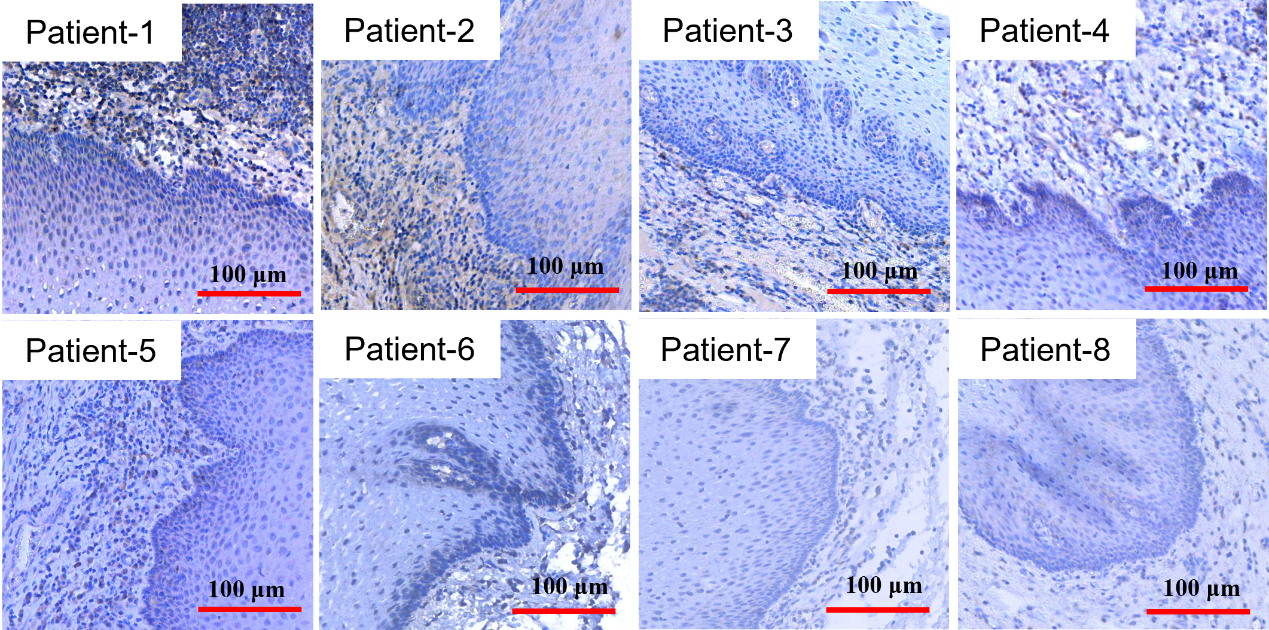


**Additional File 4. CD22 expression in ESCC patients' corresponding para-cancerous tissue**

Patient 1 to patient 8: CD22 negative-expression in ESCC patients’ para-cancerous tissues.

Scale bar, 100 μm

**Additional File 5. Expression of CD22 in ESCC cell lines**


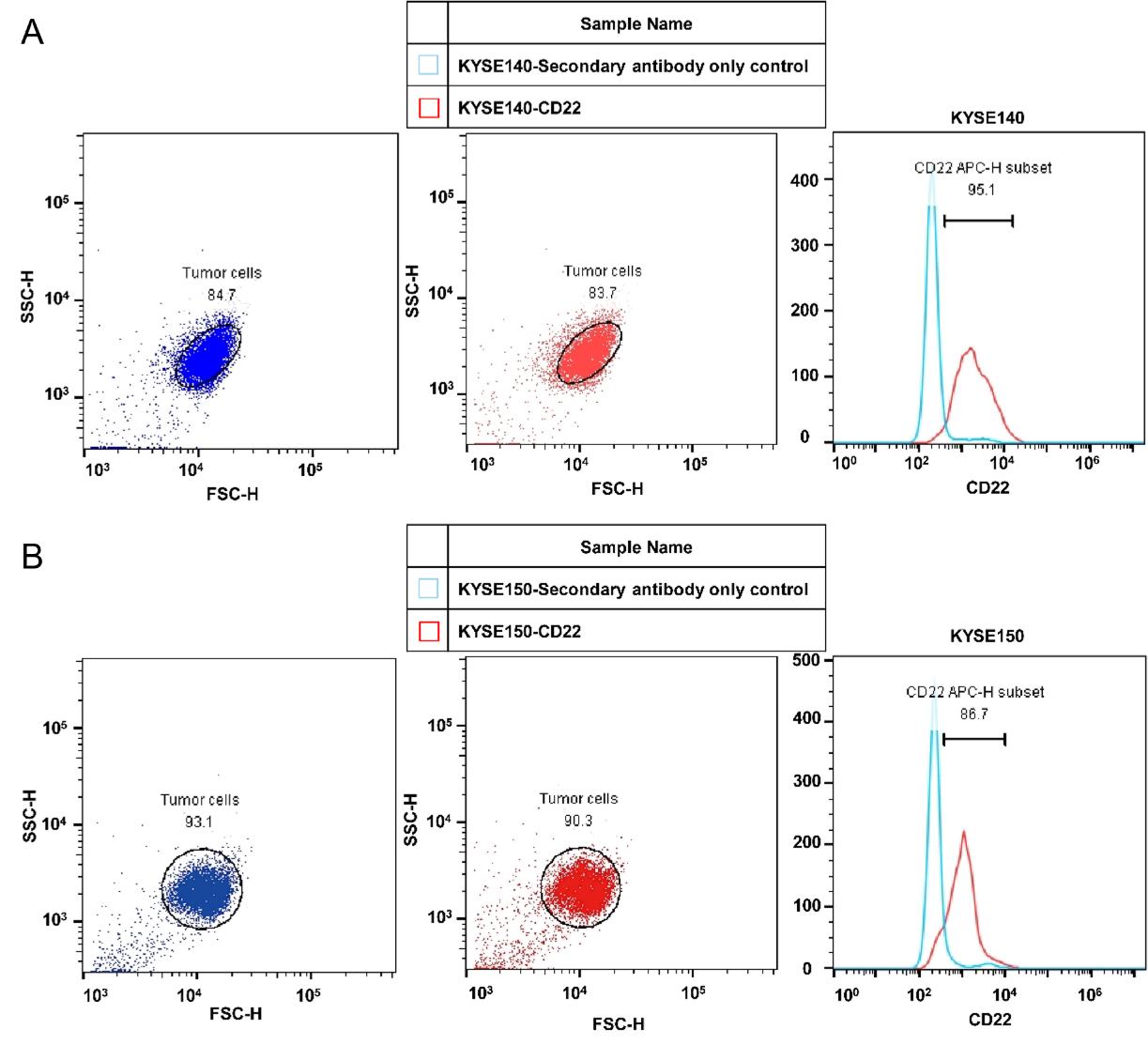


**Additional File 5. Expression of CD22 in ESCC cell lines**

A. Expression of CD22 in KYSE140 cells and its gating strategy

B. Expression of CD22 in KYSE150 cells and its gating strategy
