# Supplementary figures and images for: CD22 is a potential target of CAR-NK cell therapy for esophageal squamous cell carcinoma
Source: J Transl Med. 2023 Oct 10;21:710. doi: 10.1186/s12967-023-04409-8 (PMC10563326; doi:10.1186/s12967-023-04409-8)

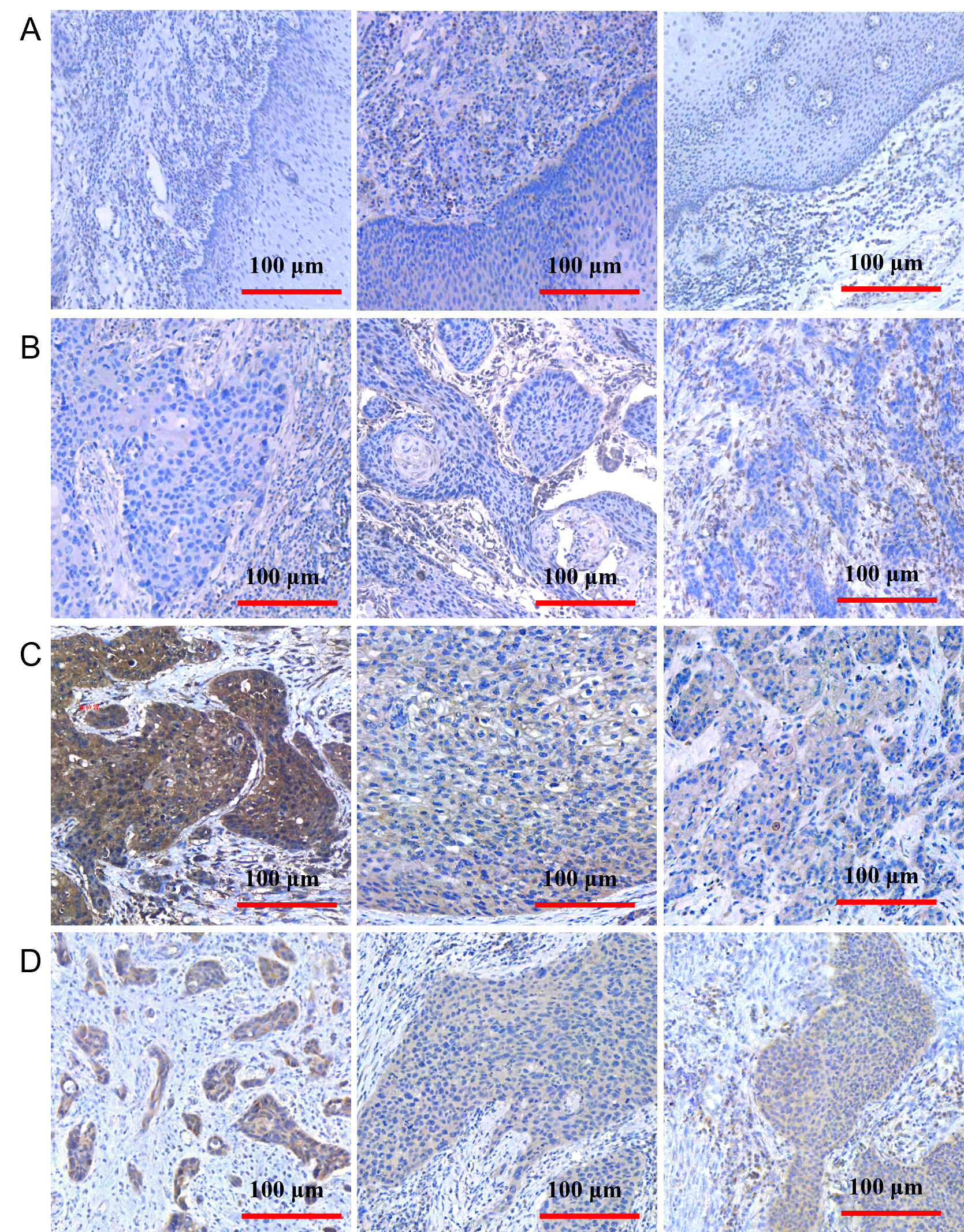

Supplement: Supplementary file 2 — Additional file 2. Expression of CD22 in ESCC tissues. [file 12967_2023_4409_MOESM2_ESM.tif]

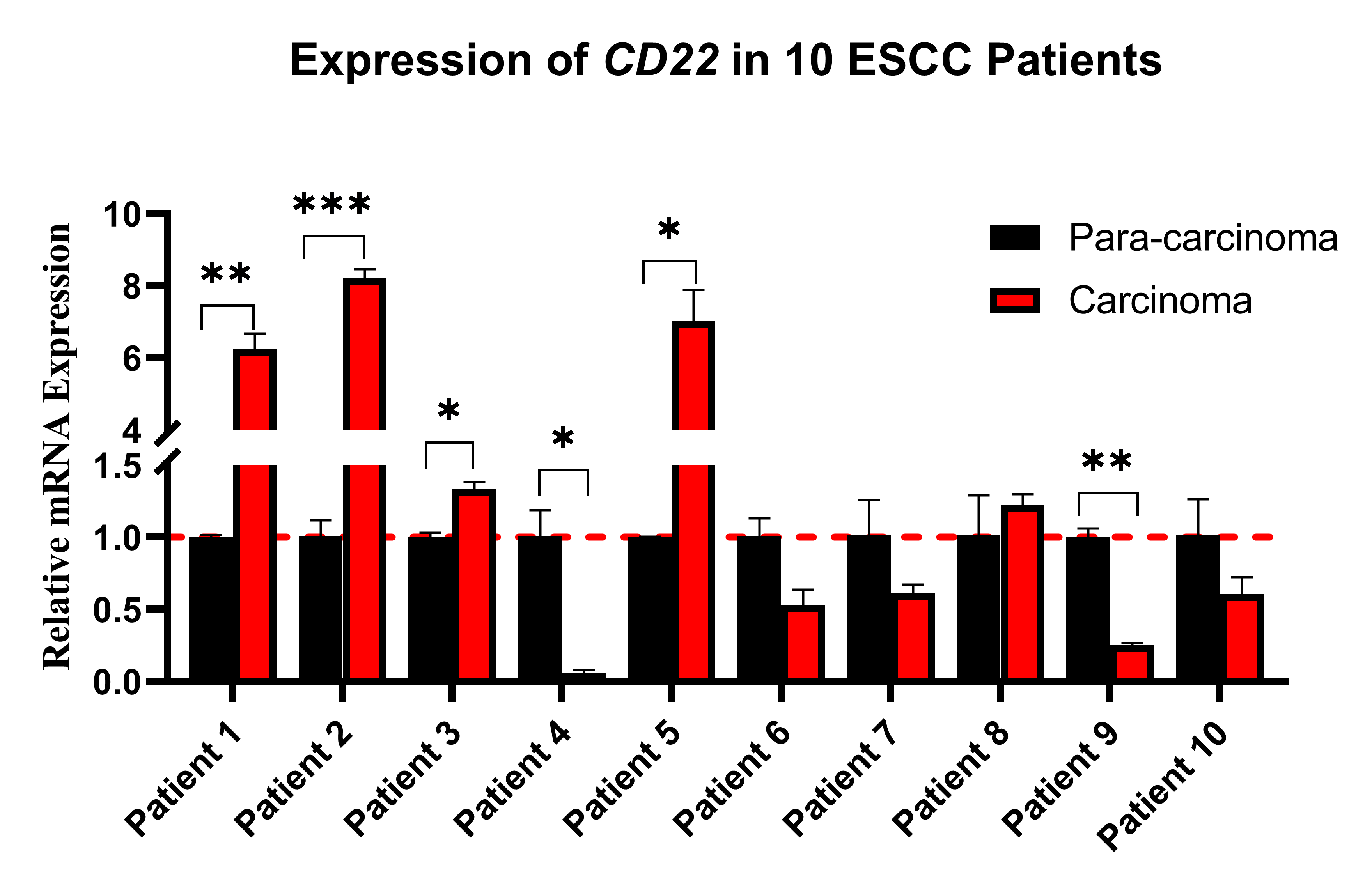

Supplement: Supplementary file 3 — Additional file 3. Expression of CD22 in ten ESCC patients. [file 12967_2023_4409_MOESM3_ESM.tif]

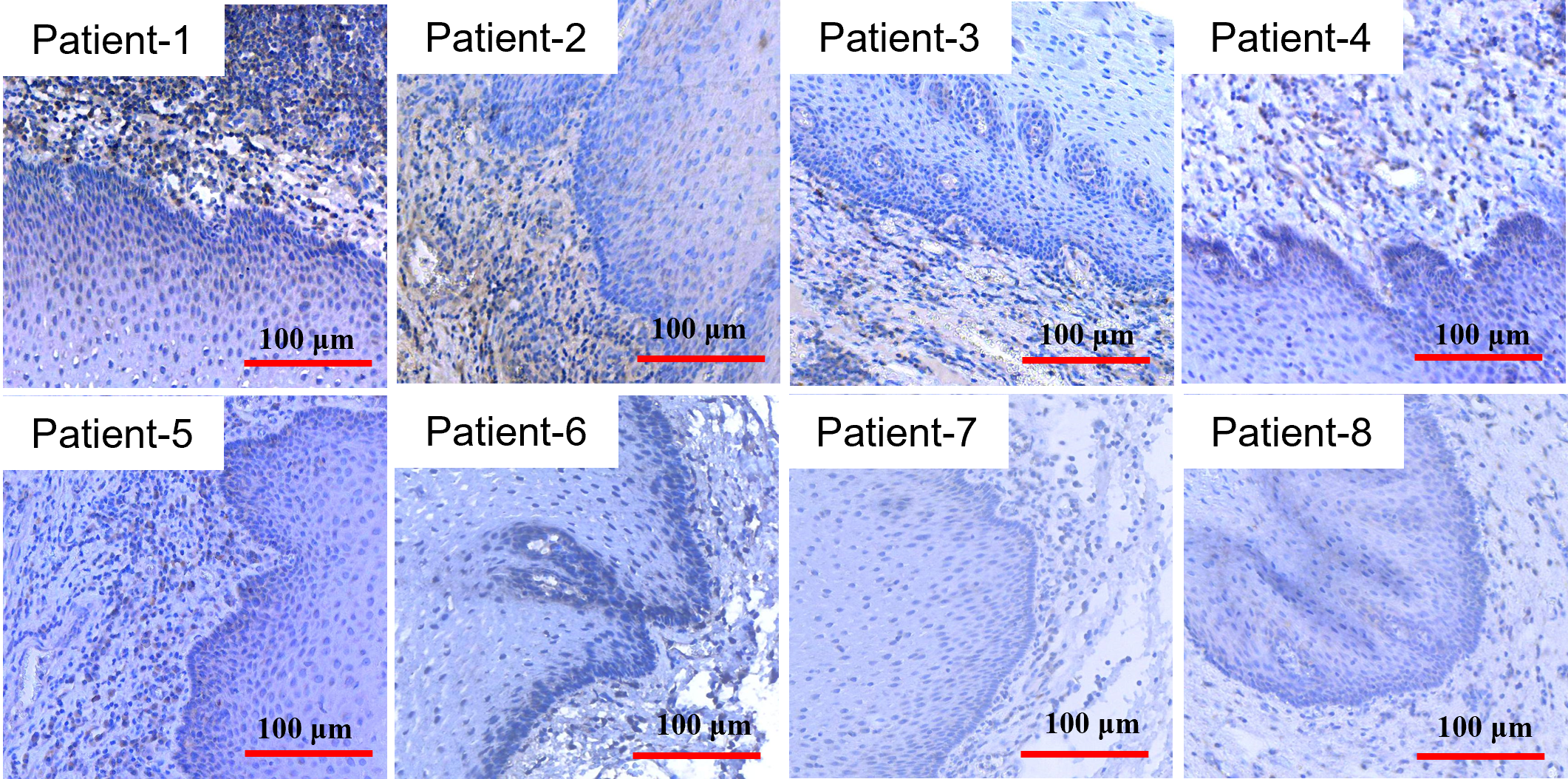

Supplement: Supplementary file 4 — Additional file 4. CD22 expression in ESCC patients' corresponding para-cancerous tissue. [file 12967_2023_4409_MOESM4_ESM.tif]

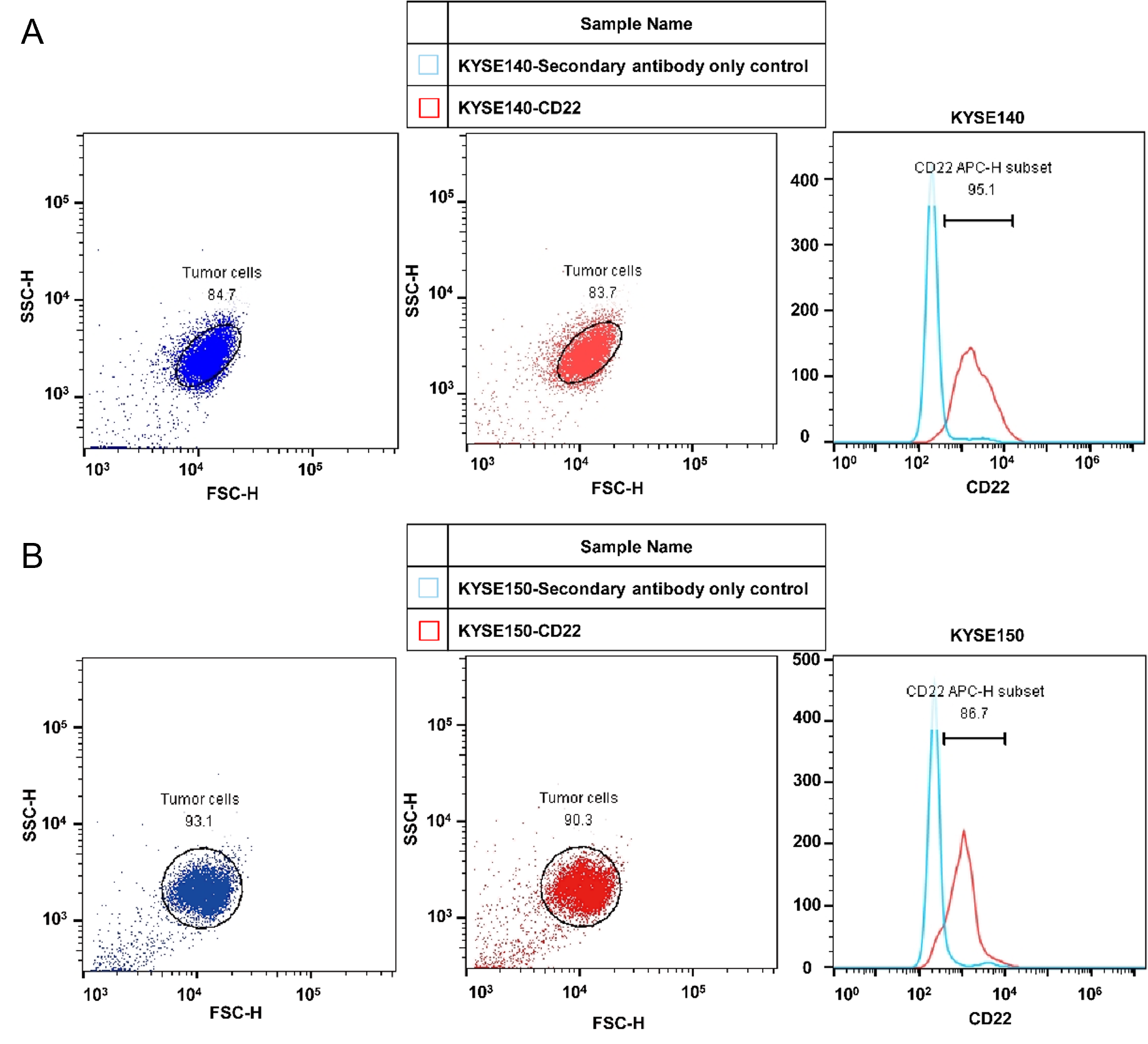

Supplement: Supplementary file 5 — Additional file 5. Expression of CD22 in ESCC cell lines. [file 12967_2023_4409_MOESM5_ESM.tif]
